# A Novel Role for IκBζ in the Regulation of IFNγ Production

**DOI:** 10.1371/journal.pone.0006776

**Published:** 2009-08-26

**Authors:** Raquel M. Raices, Yashaswini Kannan, Vedavathi Bellamkonda-Athmaram, Sudarshan Seshadri, Huating Wang, Denis C. Guttridge, Mark D. Wewers

**Affiliations:** 1 The Ohio State University, Davis Heart and Lung Research Institute, Columbus, Ohio, United States of America; 2 The Ohio State University, Department of Molecular Virology, Immunology & Medical Genetics, Columbus, Ohio, United States of America; New York University School of Medicine, United States of America

## Abstract

IκBζ is a novel member of the IκB family of NFκB regulators, which modulates NFκB activity in the nucleus, rather than controlling its nuclear translocation. IκBζ is specifically induced by IL-1β and several TLR ligands and positively regulates NFκB-mediated transcription of genes such as IL-6 and NGAL as an NFκB binding co-factor. We recently reported that the IL-1 family cytokines, IL-1β and IL-18, strongly synergize with TNFα for IFNγ production in KG-1 cells, whereas the same cytokines alone have minimal effects on IFNγ production. Given the striking similarities between the IL-1R and IL-18R signaling pathways we hypothesized that a common signaling event or gene product downstream of these receptors is responsible for the observed synergy. We investigated IκBζ protein expression in KG-1 cells upon stimulation with IL-1β, IL-18 and TNFα. Our results demonstrated that IL-18, as well as IL-1β, induced moderate IκBζ expression in KG-1 cells. However, TNFα synergized with IL-1β and IL-18, whereas by itself it had a minimal effect on IκBζ expression. NFκB inhibition resulted in decreased IL-1β/IL-18/TNFα-stimulated IFNγ release. Moreover, silencing of IκBζ expression led to a specific decrease in IFNγ production. Overall, our data suggests that IκBζ positively regulates NFκB-mediated IFNγ production in KG-1 cells.

## Introduction

We previously showed that the Interleukin-1 (IL-1) family members, IL-1β and IL-18, synergize with tumor necrosis factor-α (TNFα) for interferon-γ (IFNγ) production in the human acute myeloid leukemic KG-1 cell line [1]. IL-1β and IL-18 signal via the Interleukin-1 receptor (IL-1R) and IL-18R, respectively, both of which belong to the IL-1R family and the interleukin-1R/Toll-like receptor (IL-1R/TLR) superfamily [2]–[5]. Members of the IL-1R/TLR family share a cytoplasmic domain known as the Toll/interleukin-1 receptor (TIR) domain and recruit similar adaptor proteins, such as MyD88. Due to these and other similarities, the signaling pathways downstream of IL-1Rs and TLRs lead to similar outcomes, such as the activation of NFκB and MAPKs.

Although the IL-1β and TNFα receptors (IL-1R and TNFR) belong to different families, their signaling pathways utilize similar adaptor molecules, such as TRAFs, and lead to the activation of NFκB and MAPKs [6]–[8]. Therefore, many of the genes induced by IL-1β and TNFα overlap and the two cytokines lead to similar biological effects. However, induction of certain genes, such as neutrophil geletinase-associated lipocalin (NGAL)/lipocalin-2 [9], human β-defensin 2 (hBD2) [10]–[14], extracellular matrix metalloprotease 3 (MMP-3) [15] and IL-6 [16]–[18], is specific for IL-1β. In the same way, expression of other genes, such as complement factor H, is specific for the TNFα signaling pathway [17]. Moreover, expression of the novel member of the IκB family of NFκB regulators, IκBζ, has been shown to be specific to the IL-1R/TLR pathway (e.g. upon IL-1α/β, LPS stimulation), not the TNFα pathway [2]–[5], [19]–[24].

IκBζ expression is immediately induced upon stimulation with TLR ligands and IL-1β [19]–[31]. Moreover, IκBζ is essential for NFκB-mediated induction of genes encoding for proteins such as IL-6, NGAL, hBD2, IL-12 p40, and granulocyte-macrophage colony-stimulating factor (GM-CSF) [21], [24], [26], [30], [32]–[36], and for the suppression of E-selectin expression [33]. IκBζ has been shown to positively and negatively regulate NFκB-mediated transcription by binding to the p50 subunit of NFκB dimers [19], [21], [24], [26], [29], [30], [32]–[34], [37]. This is in contrast to other IκB members, such as IκBα/β, which are mainly found in the cytosol and modulate NFκB nuclear translocation. IκBζ is most homologous to the nuclear IκB protein, Bcl-3 [19], [23], [26], [29], which also regulates NFκB-mediated transcription as a binding co-factor [38]–[50].

We recently showed that both, IL-1β and IL-18, synergize with TNFα for IFNγ production in KG-1 cells [1]. Given the similarities between the IL-1R and IL-18R signaling pathways, we hypothesized that a common event downstream of these two receptors is crucial for the observed synergy between IL-1β/IL-18 and TNFα for IFNγ production. Even though both, the IL-1R and IL-18R, belong to the IL-1R/TLR superfamily, and IκBζ is specifically induced upon stimulation with several IL-1R/TLR ligands, IκBζ expression has not been investigated in response to IL-18 stimulation. Therefore, we analyzed IκBζ expression in KG-1 cells upon IL-18 and IL-1β stimulation, and the role of IκBζ in IFNγ production in response to combined IL-1β/IL-18 and TNFα stimulation. Our results indicate that stimulation with IL-1β and/or IL-18 results in moderate levels of IκBζ production, while TNFα has no effect. However, when combined with IL-1β or IL-18, TNFα strongly enhances IκBζ protein expression. Moreover, NFκB inhibition, as well as silencing of IκBζ expression, resulted in decreased IL-1β/IL-18/TNFα-induced IFNγ production. Furthermore, IL-1R and IL-18R expression analysis indicated that the observed synergy may take place at the receptor level in the case of IL-18 and TNFα, but not IL-1β and TNFα combined stimulation. In summary, our findings indicate that stimulation with the IL-1 cytokines, IL-1β and IL-18, in combination with TNFα results in synergistic KG-1 IFNγ production in an IκBζ/NFκB dependent manner.

## Methods

### Reagents

Purified *Escherichia coli* (E. coli) LPS (serotype 0111:B4) was obtained from Axxora (San Diego, CA) and cell culture tested ATP disodium salt from Sigma-Aldrich (St. Louis, MO). Anti-human IL-8 ELISA capture monoclonal Ab (clone 6217), IL-18 monoclonal Ab (clone 159-12B), IL-18 receptor (IL-18R) monoclonal Ab (clone 70625), and human rIL-1β, rIL-18 and rIL-8 were purchased from R&D Systems (Minneapolis, MN). An IκBα polyclonal Ab was obtained from Upstate (Billerica, Massachusetts), actin monoclonal Ab (clone C4) from MP Biomedicals (Solon, OH), p50 monoclonal (clone E-10) Ab and p65 polyclonal Ab from Santa Cruz (Santa Cruz, CA), lamin B1 polyclonal Ab from Abcam (Cambridge, MA), TNFα capture monoclonal Ab (clone 2C8) from Advanced Immunochemical (Long Beach, CA), IL-8 ELISA detection polyclonal Ab from Endogen (Rockford, IL), rTNFα from Knoll Pharmaceuticals (Whippany, NJ), Interleukin-1 receptor antagonist (IL-1ra) from Amgen (Thousand Oaks, CA), and IFNγ and IL-6 ELISA kits from eBioscience (San Diego, CA). Rabbit anti-serum against IκBζ and IL-1β and pre-immune serum were developed in our laboratory. The NFκB inhibitor, JSH23, was purchased from Calbiochem (San Diego, CA). A mixture of three different small interfering RNA (siRNA) oligonucleotides against IκBζ and three different scrambled siRNA oligonucleotides were purchased from Dharmacon RNA Technologies (Lafayette, CO). The cell line nucleofector kit R was purchased from Amaxa (Gaithersburg, MD).

### Cell Culture

KG-1 cells (American Type Culture Collection, ATCC; Manassas, VA) were maintained in RPMI 1640 (Mediatech Inc, Herndon, VA), supplemented with 20% FBS (Atlas Biologicals, Fort Collins, CO) and 1% penicillin/streptomycin in a 37°C humidified incubator with 5% CO_2_. Peripheral blood monocytes were isolated from human blood by density gradient centrifugation using lymphocyte separation media (Cell Grow, Media Tech, Herndon, VA) followed by CD14 positive selection (Miltenyi Biotec, Auburn, CA), which yields>95% purity as determined by FACS. Isolated monocytes were cultured at 10^6^/ml or at 12.5×10^6^/ml in RPMI 1640 (Cambrex, East Rutherford, New Jersey), supplemented with 5% FBS (<0.0005 EU/ml) (HyClone, Logan, UT) and 1% penicillin/streptomycin (GIBCO, Grand Island, NY), in a 37°C humidified incubator with 5% CO_2_. Monocytes were stimulated, or not, with E. coli LPS (10 ng/ml) for 4 h and ATP (5 mM) for the last 15 min of LPS stimulation, or with LPS or ATP alone. The supernatants (conditioned media) from monocytes cultured at 10^6^/ml were harvested by centrifugation (5 min; 3 600 rpm) and used to stimulate KG-1 cells.

KG-1 cells were plated at a final cell density of 10^6^/ml and incubated with test samples (monocyte conditioned media, 1/3 of the final volume) or recombinant proteins (10 ng/ml each). In selected experiments, neutralizing agents for IL-1β (IL-1β Ab – clone 2805, IL-1ra), IL-18 (IL-18 Ab – clone 125-2H, or IL-18R Ab – clone 70625), and/or TNFα (TNFα Ab – clone 2C8) were used at the indicated concentrations to neutralize the activities of rIL-18, rIL-1β and/or rTNFα, or the same endogenous cytokines present in the monocyte conditioned media, prior to incubation with KG-1 cells. Alternatively, the neutralizing agents, IL-1ra and IL-18R Ab, were used to neutralize IL-18 or IL-1 receptors prior to addition of rIL-18, rIL-1β, or monocyte conditioned media, to the KG-1 cells. KG-1 supernatants and cells were harvested at various time points for subsequent ELISA and Western blot analysis. In selected experiments, cells were treated with the NFκB inhibitor, JSH-23 (30 µM) (an NFκB nuclear translocation inhibitor), in combination with rIL-1β, rIL-18 and rTNFα for 24 h. KG-1 cells were harvested at 12 h for subsequent nuclear extraction and Western blot analysis. KG-1 supernatants were harvested at 24 h for IL-6 and IFNγ ELISA.

### Western blotting

KG-1 cells were lysed in a 60 mM Tris-HCl (pH 6.8), 2% SDS buffer. Total cell extracts were sonicated and spun (5 min; 10 000 rpm, R.T.) to remove cell debris. KG-1 nuclear/cytosolic extracts were also analyzed by Western blotting. Protein concentration in total, nuclear or cytosolic extracts was estimated using the Bio-Rad Dc protein Lowry assay (Bio-Rad). Samples were boiled in Laemmli's buffer for 5 min or heated at 70°C for 10 min in NuPAGE Sample Reducing Agent (Invitrogen). 10–40 µg of total protein were loaded per well on pre-cast 10% Tris-Glycine or 7% Tris-Acetate SDS-PAGE gels and transferred to a PVDF or nitrocellulose membrane. Membranes were blocked with 10% nonfat milk (Carnation, Nestle) in 25 mM Tris-HCl (pH 7.5), 150 mM NaCl, and 0.05% Tween for 1 h at R.T. The membranes were probed with the indicated primary Abs, followed by peroxidase-conjugated secondary antibodies. Protein bands were visualized by chemiluminescence (GE Healthcare).

### ELISA

Sandwich ELISAs were used to measure cytokine release in the supernatants of KG-1 cells.

### Flow Cytometry

KG-1 cells (10^6^/ml) were stimulated, or not, with the indicated combinations of rIL-1β, rIL-18 and rTNFα (10 ng/ml each) for 24 h. Cells were Fc-blocked by treating with 1 µg of human IgG/10^5^ for 15 min at R.T. Cells (10^5^/25 µl*reaction) were transferred to a 5 ml tube. Phycoerythrin (PE)-conjugated anti-IL-18Rα or fluorescein (FITC)-conjugated anti-IL-1R1 reagent (10 µl of each per reaction) were added to the cells. Cells were incubated for 30 min at 4°C, washed twice with 1×PBS and re-suspended in 1×PBS (10^5^/200 µl) for flow cytometric analysis. As controls, cells were also treated with phycoerythrin-labeled murine IgG_1_ and fluorescein-labeled goat IgG.

### Nuclear/Cytosolic extraction

KG-1 cells (10^6^/ml) were stimulated, or not, with rIL-1β, rIL-18, rTNFα (10 ng/ml each), or a combination of this cytokines for the indicated time points. Cells were washed twice in 1×PBS and gently re-suspended in cold Buffer A (10 mM HEPES, pH 7.9; 10 mM KCl; 0.1 mM EDTA; 0.1 mM EGTA; 1 mM dithiothreitol [DTT]; 1×Complete Mini protease inhibitor cocktail, Roche) at 400 µl/0.5−1×10^6^ cells. Cells were allowed to swell for 15 min. 10% Nonidet NP-40 was added to the solution (25 µl per 400 µl). Samples were vortexed for 10 sec and centrifuged for 30 sec (4°C, 13 200 rpm). Supernatants containing cytosolic contents were transferred to fresh tubes containing an equal volume of Buffer B (10 mM Tris-HCl, pH 7.5; 7 M urea; 1% SDS; 0.3 M NaAc; 20 mM EDTA) and stored immediately at −20°C. The pellets containing the nuclear contents were re-suspended in cold Buffer C (20 mM HEPES, pH 7.9; 0.4 M NaCl; 1 mM EDTA; 1 mM EGTA; 1 mM dithiothreitol (DTT); 1×Complete Mini protease inhibitor cocktail, Roche) at 50 µl/0.5−1×10^6^ cells. The samples were vigorously shook for 15 min at 4°C on a shaking platform and then centrifuged for 5 min (4°C, 13 200 rpm). The supernatants with the nuclear contents were stored at −20°C. Nuclear and cytosolic extracts were subsequently analyzed for protein concentration using the Bio-Rad Dc protein Lowry assay (Bio-Rad). Nuclear and cytosolic extracts were then prepared for Western blot analysis.

### Small interfering RNA

KG-1 cells (2×10^6^/ml) were nucleofected following the protocol for KG-1 cell nucleofection provided with the cell line nucleofector kit R from Amaxa (Gaithersburg, MD) with a mixture of 3 different small interfering RNA (siRNA) oligonucleotides against IκBζ or 3 different scrambled siRNA oligonucleotides (3 µg per 2×10^6^ cells). After 2 h, cells were stimulated with a combination of rIL-1β, rIL-18 and rTNFα (10 ng/ml each). Cells and supernatants were harvested at 24 h for subsequent RNA and protein analysis (qPCR and Western blot, respectively).

### Quantitative PCR (qPCR)

KG-1 cells (10^6^/ml) were lysed in TRIzol reagent (Invitrogen Life Technologies) and mRNA was extracted and converted to cDNA using the Thermoscript RT-PCR system (Invitrogen Life Technologies). qPCR was performed using specific primers for IFNγ, IL-6 and IL-8. Values were normalized to two house-keeping genes, CAP-1 and GAPDH.

### Statistical analysis

Data are presented mean±S.E.M. from≥3 independent experiments. Comparisons were done by paired t-test with *p*<0.05 defined as statistically significant.

## Results

### IL-1β and IL-18, but not TNFα, induce IκBζ protein expression in KG-1 cells

We have recently demonstrated that IL-1R and IL-18R agonists synergize with TNFα for IFNγ production in KG-1 cells [1]. The IL-1R, IL-18R and TLRs all belong to the IL-1R/TLR superfamily [2]. Because expression of the IκB protein family member, IκBζ, is known to be induced downstream of the IL-1R and TLRs [19]–[31], we chose to evaluate its potential role in IFNγ production in response to IL-1β/IL-18 and TNFα combined stimulation. We analyzed induction of IκBζ protein expression in response to IL-1β and IL-18, with the idea that IκBζ may be the common factor downstream of the IL-1R and IL-18R, responsible for the observed synergy between IL-1β/IL-18 and TNFα for IFNγ production [1].

**Figure 1:**
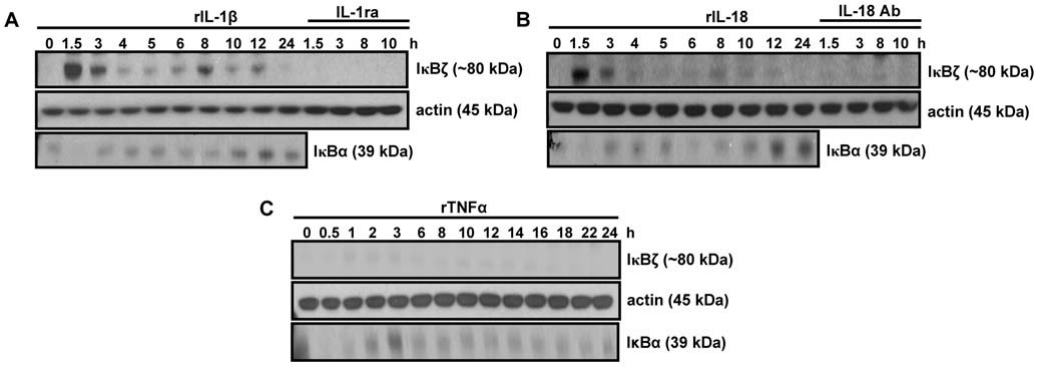
IκBζ protein expression in KG-1 cells in response to IL-1β, IL-18 and TNFα stimulation. KG-1 cells (10^6^/ml) were stimulated with rIL-1β (A), rIL-18 (B), or rTNFα (C) (10 ng/ml each) for the indicated time points. At selected time points, the cells were incubated with rIL-1β and rIL-18 in the presence of IL-1ra (100 µg/ml) or IL-18 Ab (2 µg/ml), respectively. Protein-matched total cell extracts were analyzed by Western blotting using anti-serum against IκBζ, IkBα Ab and actin Ab. Results are representative of at least 3 separate experiments.

In this context, KG-1 cells were stimulated with rIL-1β, rIL-18 and rTNFα for various time points, with and without co-addition of IL-1ra or IL-18 Ab. Total cell extracts were analyzed for IκBζ protein expression by Western blotting. Results indicated that both rIL-1β and rIL-18, but not rTNFα, induced IκBζ expression in KG-1 cells (Fig. 1A–C). The lack of rTNFα-mediated IκBζ expression was not due to lack of biological activity of rTNFα, as judged by the modulation (degradation and *de novo* protein synthesis) of IκBα expression upon rTNFα stimulation (Fig. 1C). The finding that IL-1β, as well as IL-18, both induce IκBζ protein expression supports the hypothesis that IκBζ may be the common factor downstream of the IL-1R and IL-18R that allows for synergy between IL-1 cytokines (IL-1β and IL-18) and TNFα for IFNγ production in KG-1 cells.

### TNFα enhances IL-1β/IL-18-mediated IκBζ expression

Stimulation with TNFα alone does not lead to IκBζ expression (Fig. 1C) [19]–[21], [24]. However, TNFα has been shown to induce IκBζ transcription to a greater extent than IL-1β and LPS [20]. In contrast, upon actinomycin D treatment, the half-life of ectopically expressed IκBζ mRNA was prolonged with IL-1β and LPS, but unaffected by TNFα. Therefore, even though TNFα has strong IκBζ transcriptional activity, IL-1/LPS may provide additional mRNA stabilization (absent with solo TNFα stimulation) leading to subsequent protein expression. Based on this information, we hypothesized that TNFα may enhance IL-1β/IL-18-mediated IκBζ protein expression by providing strong transcriptional activation, even though by itself it does not lead to IκBζ protein expression.

**Figure 2:**
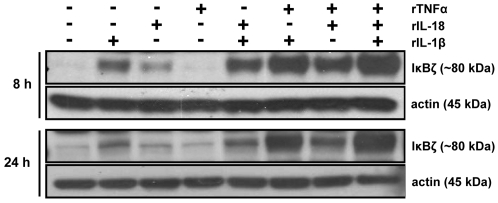
TNFα enhances IL-1β/IL-18-induced IκBζ protein expression in KG-1 cells. KG-1 cells (10^6^/ml) were stimulated with rIL-1β, rIL-18, rTNFα (10 ng/ml each), or different combinations of these cytokines for 8 and 24 h. Protein-matched total cell extracts were analyzed by Western blotting using anti-serum against IκBζ and actin Ab.

**Figure 3:**
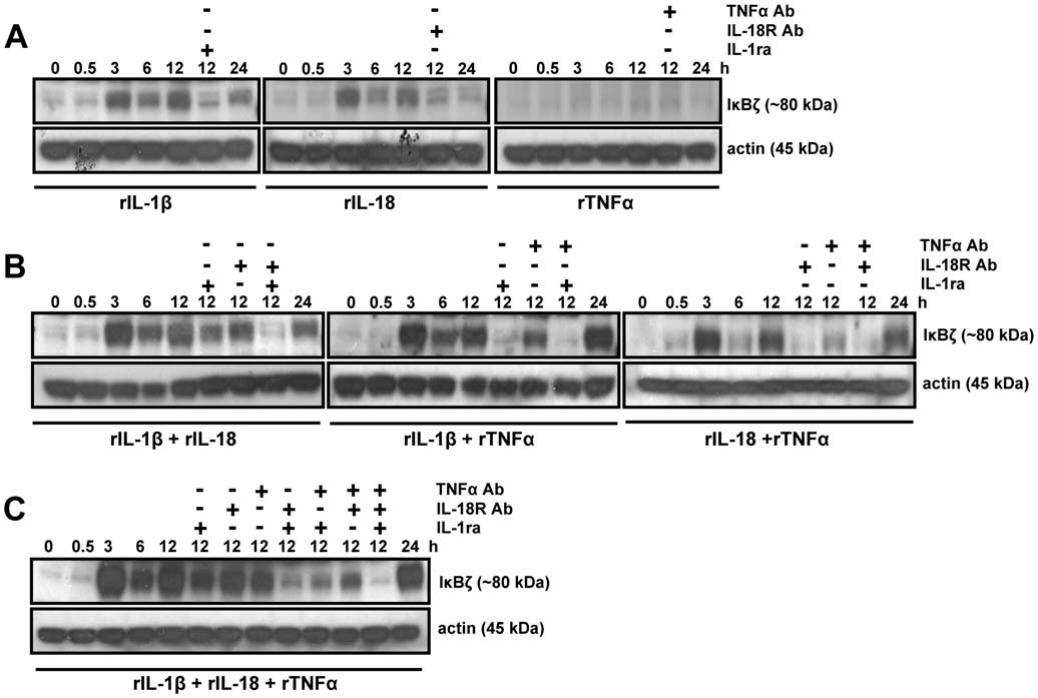
The synergistic effect of TNFα and IL-1β/IL-18 on IκBζ protein expression is partially suppressed with a TNFα-specific Ab, but completely blocked with IL-1β and/or IL-18 neutralization. KG-1 cells (10^6^/ml) were stimulated with rIL-1β, rIL-18, rTNFα (10 ng/ml each) (A), or the indicated combinations of these cytokines (B and C) for the indicated time points. At selected time points, the cells were incubated with the recombinant proteins in the presence of IL-1ra (100 µg/ml), IL-18R Ab (10 µg/ml), TNFα Ab (10 µg/ml), or different combinations of these neutralizing agents. Protein-matched total cell extracts were analyzed by Western blotting using anti-serum against IκBζ and actin Ab. Results are representative of at least 3 separate experiments.

To test this hypothesis, KG-1 cells were stimulated with rTNFα alone and in combination with rIL-1β, rIL-18, or both, for 8 and 24 h. Total cell extracts were analyzed for IκBζ protein expression by Western blotting. Recombinant TNFα enhanced rIL-1β- and rIL-18-mediated IκBζ protein expression at both time points (Fig. 2). We then analyzed the kinetics of KG-1 IκBζ protein expression in response to different combinations of rIL-1β, rIL-18 and rTNFα, with and without co-addition of IL-1ra, IL-18R Ab, TNFα Ab, or different combinations of these neutralizing agents. Interestingly, IκBζ protein expression followed an oscillating pattern (Fig. 3), which is typical of IκB proteins, such as IκBα. Moreover, it was evident that the observed induction of IκBζ protein upon stimulation with rTNFα combined with rIL-1β, rIL-18, or both, was in part due to rTNFα, since the induction was only partially suppressed with a TNFα neutralizing Ab (Fig. 3B and C). The remaining IκBζ protein expression after TNFα neutralization was likely due to IL-1β and/or IL-18. As expected, neutralization with IL-1ra or IL-18R Ab, resulted in complete inhibition of IκBζ expression, indicating that TNFα by itself has no IκBζ inducing activity. Interestingly, rIL-1β, rIL-18 or the combination of these cytokines (at a dose of 10 ng/ml) results in minimal amounts of IFNγ production by KG-1 cells, despite their ability to induce IκBζ protein expression [1]. Therefore, the levels of IL-1β/IL-18-induced IκBζ protein may either not be sufficient for significant IFNγ production (in the absence of TNFα stimulation), or may require an additional TNFα-induced factor for activation of the IFNγ promoter.

### The conditioned media from LPS/ATP-stimulated monocytes induces IκBζ expression in an IL-1β-dependent, but IL-18-independent manner

**Figure 4:**
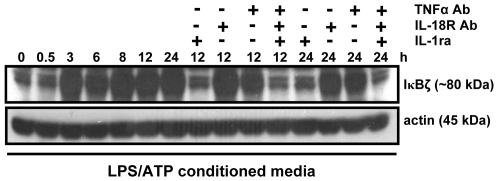
The conditioned media from LPS/ATP-stimulated monocytes induces IκBζ expression in an IL-1β/TNFα-dependent, but IL-18-independent manner. KG-1 cells (10^6^/ml) were incubated with conditioned media from monocytes (10^6^/ml) that were stimulated with LPS (10 ng/ml, 4 h) and ATP (5 mM, last 15 min) for the indicated time points. For selected time points, the cells were incubated with the conditioned media in the presence of IL-1ra (100 µg/ml), IL-18R Ab (10 µg/ml), TNFα Ab (10 µg/ml), or different combinations of these neutralizing agents. Protein-matched total cell extracts were analyzed by Western blotting using anti-serum against IκBζ and actin Ab. Results are representative of 3 separate experiments.

We have recently shown that the conditioned media from LPS/ATP-treated monocytes induces IFNγ release by KG-1 cells and that this induction is due to the synergistic effect of IL-1β and TNFα, and independent of IL-18 [1]. Herein, we incubated KG-1 cells with conditioned media from LPS/ATP-stimulated monocytes for various time points, with and without co-addition of IL-1ra, IL-18R Ab, TNFα Ab, or different combinations of these neutralizing agents, and analyzed IκBζ protein expression. The monocyte conditioned media induced IκBζ protein expression in an IL-1β-dependent, but IL-18-independent manner (Fig. 4). This finding correlates with our previous observation that endogenous IL-18 present in the conditioned media from LPS/ATP-stimulated monocytes does not induce IFNγ production by KG-1 cells [1]. The lack of IL-18 IFNγ inducing activity in the supernatants of LPS/ATP-stimulated monocytes may be due to low levels of IL-18 being released or to IL-18 being bound to its biological inhibitor, IL-18BP [1].

### TNFα upregulates IL-18R, not IL-1R expression

**Figure 5:**
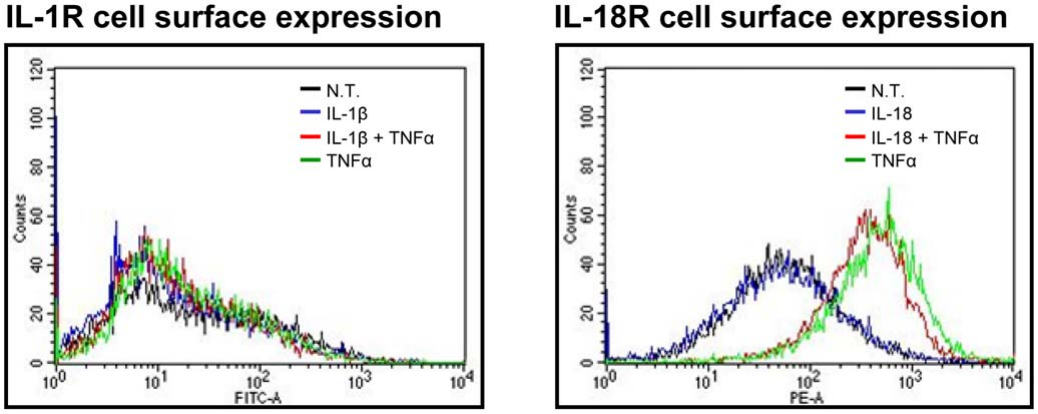
Effect of TNFα treatment on KG-1 IL-18R and IL-1R surface expression. KG-1 cells (10^6^/ml) were incubated with the indicated combinations of rIL-1β, rIL-18, rTNFα (10 ng/ml each) for 24 h. Cells were stained with IL-18R-PE and IL-1R-FITC followed by flow cytometry analysis. Results are representative of 3 separate experiments.

TNFα has been shown to upregulate expression of the IL-18R in KG-1 cells [51]–[57]. Therefore, TNFα may synergize with IL-1β in a similar manner for IκBζ and IFNγ production – by upregulating surface expression of the IL-1R. In order to explore this possibility, KG-1 cells were treated with the indicated combinations of rIL-1β, rIL-18 and rTNFα for 24 h. TNFα treatment resulted in upregulation of IL-18R, not IL-1R expression, as determined by flow cytometry (Fig. 5). Therefore, a signaling event(s) downstream of the IL-1R and IL-18R, rather that TNFα-mediated receptor upregulation, is likely to be crucial for the observed synergy between IL-1 cytokines and TNFα for IκBζ and IFNγ production. Moreover, the greater IFNγ production in response to rIL-18 in combination with rTNFα, compared to rIL-1β in combination with rTNFα [1], may be explained by additional TNFα-mediated upregulation of the IL-18R.

### IκBζ protein localizes to the nucleus

**Figure 6:**
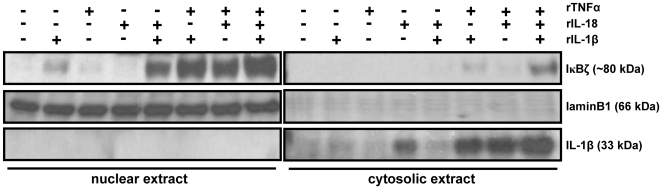
IκBζ mainly localizes to the nucleus. KG-1 cells (10^6^/ml) were stimulated with rIL-1β, rIL-18, rTNFα (10 ng/ml each), or different combinations of these cytokines for 8 h. Cells were harvested for subsequent cytosol and nuclear extraction. Protein-matched nuclear and cytosolic extracts were then analyzed by Western blotting with anti-serum against IκBζ, laminB1 Ab (nuclear marker) and IL-1β Ab (cytosolic marker). Results are representative of 3 separate experiments.

IκBζ protein has been shown to localize to the nucleus in most cell types [19], [23], [26], [28], [34]. However, IκBζ has also been shown to localize to the cytoplasm in B cell rich regions of immune organs, such as lymphoid follicles in the spleen [28]. In order to confirm the cellular localization of IκBζ protein in the KG-1 cell line, cells were stimulated with rIL-1β, rIL-18, rTNFα and different combinations of these cytokines for 8 h and harvested for cytosol and nuclear extraction. Results demonstrate that IκBζ protein localizes predominantly to the nucleus of KG-1 cells (Fig. 6), consistent with its role as a co-factor for NFκB-mediated transcription.

### NFκB inhibition leads to decreased IFNγ and IL-6 release

**Figure 7:**
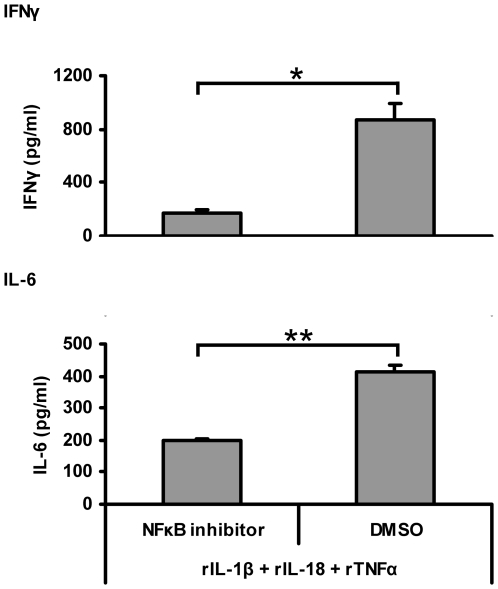
NFκB inhibition results in decreased IFNγ and IL-6 release. KG-1 cells (10^6^/ml) were incubated with the NFκB inhibitor, JSH23 (30 µM), and subsequently stimulated with a combination of rIL-1β, rIL-18 and rTNFα (10 ng/ml each). Supernatants were harvested after 24 h and analyzed for cytokine release by IL-6 and IFNγ ELISAs. Results are shown as mean±S.E.M. *, *p*<0.05; **, *p*<0.005 (n = 3).

IκBζ has been shown to act as an NFκB binding co-factor by associating with the p50 NFκB subunit. Therefore, we decided to test whether IFNγ release in KG-1 cells in response to IL-1β, IL-18 and TNFα combined stimulation is NFκB dependent. KG-1 cells were incubated with an inhibitor of NFκB nuclear translocation followed by IL-1β, IL-18 and TNFα combined stimulation. Western blot analysis with nuclear extracts indicated a reduction in p50 and p65 nuclear localization, indicative of a decrease in NFκB activity (data not shown). Moreover, IL-6 and IFNγ release were significantly reduced with NFκB inhibition (Fig. 7). Therefore, IκBζ may regulate IFNγ release in KG-1 cells in response to IL-1β, IL-18 and TNFα combined stimulation by acting as a co-factor for NFκB-mediated transcription.

### Silencing of IκBζ suppresses IFNγ and IL-6, not IL-8 production

IκBζ has been shown to either negatively or positively regulate NFκB activity depending upon the context. Genes, such as E-selectin, are negatively regulated [33], whereas genes such as IL-6 and NGAL, are positively regulated [21], [24], [26], [32]–[35]. Because IL-1β/IL-18 or TNFα blockade inhibits both IκBζ protein expression and IFNγ production in KG-1 cells, we hypothesized that IκBζ positively regulates IFNγ production.

**Figure 8:**
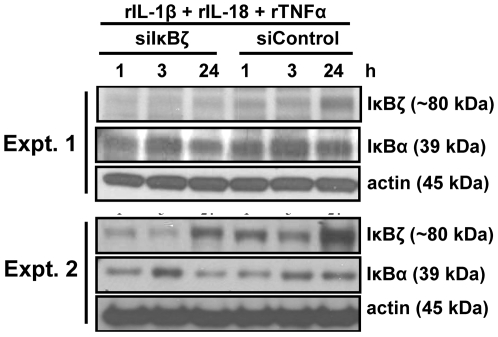
Silencing of IκBζ expression. KG-1 cells (2×10^6^/ml) were nucleofected with a mixture of 3 different small interfering RNA (siRNA) oligonucleotides against IκBζ or 3 different scrambled siRNA oligonucleotides. After 2 h, cells were stimulated with a combination of rIL-1β, rIL-18 and rTNFα (10 ng/ml each) for the indicated time points. Protein-matched total cell extracts were analyzed by Western blotting using anti-serum against IκBζ and actin Ab. Results are representative of 5 separate experiments.

**Figure 9:**
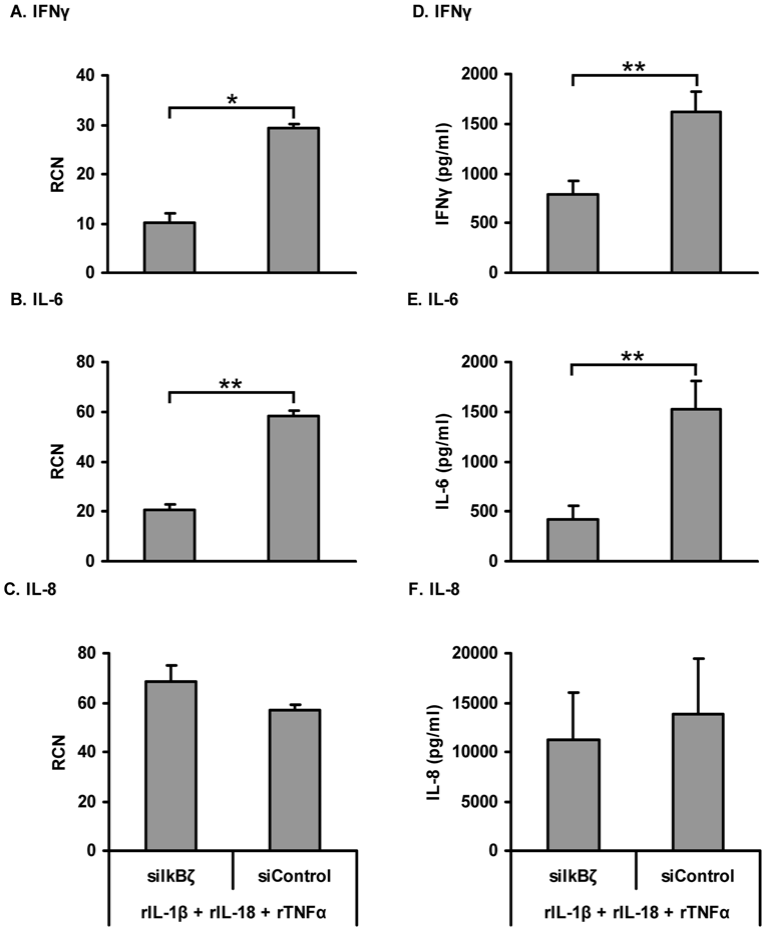
Silencing of IκBζ expression suppresses IFNγ and IL-6, not IL-8, mRNA and protein production. KG-1 cells (2×10^6^/ml) were nucleofected with a mixture of 3 different small interfering RNA (siRNA) oligonucleotides against IκBζ or 3 different scrambled siRNA oligonucleotides. After 2 h, cells were stimulated with a combination of rIL-1β, rIL-18 and rTNFα (10 ng/ml each) for 24 h. Cells were lysed for mRNA extraction. Messenger RNA (mRNA) was converted to cDNA, followed by quantitative PCR (qPCR) using primers specific for IFNγ (A), IL-6 (B) and IL-8 (C). Supernatants were harvested and analyzed for cytokine release by IFNγ (D), IL-6 (E) and IL-8 (F) ELISA. Results are shown as mean±S.E.M. *, *p*<.05; **, *p*<0.005 (A, B and C, n = 3) (D, E and F, n = 5).

In order to test this hypothesis, KG-1 cells were nucleofected with a mixture of 3 different small interfering RNA (siRNA) oligonucleotides against IκBζ or 3 different scrambled siRNA oligonucleotides. Cells were then stimulated with a combination of rIL-1β, rIL-18 and rTNFα. Western blot analysis indicated a reduction in IκBζ protein expression with anti-IκBζ siRNA delivery (Fig. 8). In order to determine the effect of IκBζ silencing on IFNγ protein production, we measured IFNγ mRNA levels and protein release in KG-1 cells upon rIL-1β, rIL-18 and rTNFα combined stimulation (Fig. 9). As a positive control, we measured IL-6 mRNA and protein release since this cytokine has been shown to be positively regulated by IκBζ [21], [26], [32], [34], [35]. As a negative control, we measured IL-8 mRNA and protein levels, which have been shown not to be regulated by IκBζ [24], [33]. Results indicated that the mRNA and protein levels of IFNγ and IL-6, but not IL-8, were significantly reduced with anti-IκBζ siRNA delivery (Fig. 9). These results implicate a role for IκBζ as a positive regulator of IFNγ production.

## Discussion

Regulation of IFNγ gene transcription involves the action of many different transcripition factors including STATs, AP-1, GATA-3, NFAT, T-bet, Eomesodermin, NFκB, NFAT, T-bet, YY-1, DREAM, ERM and SMADs. In most cases, multiple signals synergize for IFNγ production via induction of different transcription factors that act in concert to induce gene expression [58]–[61]. The combination of IL-12 and IL-18 is the most well-known example of synergy between two cytokines for IFNγ production in T cells, NK/NKT cells, B cells, macrophages and dendritic cells [62]–[70]. Synergy between IL-12 and IL-18 occurs not only at the transcription factor level via STAT4 and AP-1 activation, respectively, but also at the receptor level, with both cytokines upregulating cell surface expression of each other's receptors. Synergy for IFNγ production has also been observed with the combination of receptor crosslinking and cytokine stimulation. As an example, the combination of LY49 activating receptor crosslinking and IL-12 or IL-18 synergistically enhance IFNγ production in NK cells via the p38 MAP kinase and the ERK-dependent signal transduction pathways [71].

In general, IL-12 and IL-18 require each other for IFNγ gene expression. However, at high doses (50 ng/ml), IL-18 alone can induce IFNγ production in the human acute myeloid leukemic KG-1 cell line [1], [24]. KG-1 cells have been widely used to study IL-18-mediated signaling events leading to IFNγ expression. The responsiveness of KG-1 cells to IL-18 (in absence of IL-12) is partly due to constitutive expression of both chains of the IL-18R [72], [73], whereas primary NK and T cells require IL-12 stimulation for expression of the binding chain of the IL-18R [70], [74]–[77].

NFκB has been shown to regulate the expression of many pro-inflammatory genes, IFNγ being no exception. Two putative NFκB binding sites have been identified in the IFNγ promoter region (κBB site and CD28RE) and one in the first intron (C3) [78]. The requirement for NFκB in IFNγ gene expression appears to be contingent on the cell type and the specific stimulus. IL-18 signaling via the IL-18R leads to NFκB activation [78]–[82]. Moreover, stimulation of KG-1 cells with high doses (50 ng/ml) of IL-18 leads to IFNγ production in an NFκB-dependent manner [78].

We have previously described a novel synergistic role for the members of the IL-1 family, IL-1β and IL-18, in combination with TNFα in IFNγ production in KG-1 cells [1]. Importantly, at the dose of 10 ng/ml, the individual cytokines induced only minimal amounts of IFNγ release. Given the striking similarities between the IL-1β and IL-18 signaling pathways, we proposed that a common factor downstream of the IL-1R and IL-18R is responsible for the observed synergy between IL-1β/IL-18 and TNFα. The latter is supported by the fact that induction of the IFNγ promoter is generally mediated by multiple signals leading to activation of multiple transcription factors that synergistically induce IFNγ gene expression [58]–[61].

The novel member of the IκB family of NFκB regulators, IκBζ, is known to be induced by IL-1R/TLR ligands. Moreover, even though the TNFR signaling pathway shares some similarities with the IL-1R/TLR pathway, such as the use of TRAF adaptor molecules, TNF signaling alone does not result in IκBζ protein expression [2]–[5], [19]–[24]. Although IL-18 signals via a member of the IL-1R family, it has never been tested as an inducer of IκBζ expression. We have shown for the first time that IL-18 stimulation also leads to IκBζ protein expression in KG-1 cells.

IκBζ has been shown to positively regulate NFκB-mediated transcription of secondary response genes such as IL-6 and NGAL, as a co-factor binding to the p50 NFκB subunit [21], [24], [26], [32], [33], [35]. Moreover, NFκB has been shown to play an important role as a positive regulator of IFNγ gene expression in thymocytes, peripheral blood T lymphocytes and KG-1 cells [66], [78], [83]. Therefore, we hypothesized that IκBζ may be the common factor downstream of the IL-1R and IL-18R pathways, which allows for synergy between IL-1 cytokines and TNFα for IFNγ production in KG-1 cells.

Interestingly, we observed that TNFα enhanced IL-1β/IL-18-mediated IκBζ protein expression, even though by itself it had not effect on IκBζ protein expression. However, TNFα, IL-1β and LPS have all been shown to induce IκBζ mRNA transcription in NIH3T3 and A549 cells [20]. Importantly, TNFα stimulation alone results in strong activation of the IκBζ promoter without subsequent protein expression. Moreover, nuclear run-on analysis in NIH3T3 cells also indicates that TNFα is a stronger transcriptional activator of the IκBζ gene, compared to IL-1β or LPS. Furthermore, decay analysis of ectopically expressed IκBζ mRNA upon actinomycin D treatment, indicates that degradation of IκBζ mRNA is delayed by IL-1β and LPS stimulation, but not by TNFα stimulation. Moreover, the N-terminal half, not the C-terminal half, of the IκBζ ORF confers IL-1/LPS-mediated IκBζ mRNA stabilization. Therefore, the specificity of IL-1/LPS stimulus for IκBζ mRNA and protein induction is most likely at the post-transcriptional level and due to stabilization of IκBζ mRNA. A *cis*-element in the N-terminal half of the IκBζ gene appears to be crucial for this stabilization [20].

In support of the latter findings, the NGAL promoter has been shown to be specifically induced by IL-1β, not by TNFα [9], [21], [84], [85]. NGAL promoter activity requires NFκB activation and an intact NFκB binding site [24]. Even though IL-1β and TNFα, both induce NFκB nuclear translocation and recruitment to the NGAL promoter, only IL-1β is able to induce NGAL expression. IκBζ has been shown to be the co-factor which allows NFκB to mediate NGAL gene expression downstream of the IL-1R/TLR signaling pathway [24]. The latter was shown by IκBζ over expression in A549 cells, which rescued TNFα-induced NGAL expression. The same may apply to expression of the IL-6 gene, which at least in the KG-1 cell line (data not shown) and other cell types [16]–[18], is specific to IL-1β, not TNFα stimulation, and to other genes specifically induced downstream of the IL-1R/TLR pathway. As an additional example, hBD2 is also stimulated by IL-1β, but not TNFα stimulation in human keratinocytes [9], [84] and A549 cells [24]. Moreover, siRNA experiments have shown that IκBζ is critical for IL-1β mediated hBD2 mRNA expression in A549 cells [24].

Based on this information, we conclude that while IL-1β and IL-18 may provide the signal(s) required for IκBζ transcriptional activation, mRNA stabilization and subsequent IκBζ protein expression, TNFα may enhance IL-1β/IL-18-mediated IκBζ expression by providing strong transcriptional activation of the IκBζ gene. Moreover, TNFα stimulation of KG-1 cells provides robust binding of the p50 and p65 NFκB subunits to EMSA probes containing the NFκB binding sites present in the IFNγ promoter and first intron (data not shown), compared to weaker binding provided by IL-1β and IL-18. Therefore, robust NFκB activation provided by TNFα may also result in increased IFNγ gene expression since IκBζ regulates transcription as a co-factor for NFκB and KG-1 IFNγ production in response to IL-1/TNFα combined stimulation is NFκB dependent (Fig. 7). Thus, the synergy between IL-1β/IL-18 and TNFα may be due to their combined effects on IκBζ expression, as well as on NFκB activation. Alternatively, other transcription factors induced by TNFα may synergize with IκBζ/NFκB for IFNγ gene expression. Receptor expression analysis indicated that TNFα-mediated upregulation of IL-1/IL-18R expression did not account for the synergy between these cytokines for IκBζ production.

In summary, we have shown a positive role for IκBζ on IFNγ production in response to IL-1β, IL-18 and TNFα combined stimulation in KG-1 cells. This regulation is most likely dependent on the ability of IκBζ to regulate NFκB mediated transcription of the IFNγ gene. This finding represents a new addition to the complex and continuously growing literature on the regulation of IFNγ expression.
